# Cross-Reactivity of Antibodies against Leptospiral Recurrent Uveitis-Associated Proteins A and B (LruA and LruB) with Eye Proteins

**DOI:** 10.1371/journal.pntd.0000778

**Published:** 2010-08-03

**Authors:** Ashutosh Verma, Pawan Kumar, Kelly Babb, John F. Timoney, Brian Stevenson

**Affiliations:** 1 Department of Microbiology, Immunology and Molecular Genetics, University of Kentucky College of Medicine, Lexington, Kentucky, United States of America; 2 Gluck Equine Research Center, University of Kentucky, Lexington, Kentucky, United States of America; Institut Pasteur, France

## Abstract

Infection by *Leptospira interrogans* has been causally associated with human and equine uveitis. Studies in our laboratories have demonstrated that leptospiral lipoprotein LruA and LruB are expressed in the eyes of uveitic horses, and that antibodies directed against LruA and LruB react with equine lenticular and retinal extracts, respectively. These reactivities were investigated further by performing immunofluorescent assays on lenticular and retinal tissue sections. Incubation of lens tissue sections with LruA-antiserum and retinal sections with LruB-antiserum resulted in positive fluorescence. By employing two-dimensional gel analyses followed by immunoblotting and mass spectrometry, lens proteins cross-reacting with LruA antiserum were identified to be α-crystallin B and vimentin. Similarly, mass spectrometric analyses identified β-crystallin B2 as the retinal protein cross-reacting with LruB-antiserum. Purified recombinant human α-crystallin B and vimentin were recognized by LruA-directed antiserum, but not by control pre-immune serum. Recombinant β-crystallin B2 was likewise recognized by LruB-directed antiserum, but not by pre-immune serum. Moreover, uveitic eye fluids contained significantly higher levels of antiibodies that recognized α-crystallin B, β-crystallin B2 and vimentin than did normal eye fluids. Our results indicate that LruA and LruB share immuno-relevant epitopes with eye proteins, suggesting that cross-reactive antibody interactions with eye antigens may contribute to immunopathogenesis of *Leptospira*-associated recurrent uveitis.

## Author Summary

*Leptospira* is the most common infectious cause of uveitis, a potentially debilitating inflammation of the eye. In our earlier work, we discovered that eye fluids of uveitic horses contain high levels of antibodies directed against novel leptospiral proteins, which we named LruA and LruB (**L**eptospiral **r**ecurrent **u**veitis associated proteins **A** and **B**). Significantly, antibodies raised against LruA and LruB also recognize lens and retinal tissue. We have now identified the cross-reactive eye proteins as alpha-crystallin B, vimentin and beta-crystallin B2. We also demonstrated that ocular fluids from uveitic horses contain high levels of antibodies recognizing alpha-crystallin B, vimentin and beta-crystallin B2. These data suggest that antibodies directed against leptospiral LruA and LruB during infection can also react with eye proteins, alpha-crystallin B, vimentin and beta-crystallin B2, potentially contributing to the severity of this eye disease.

## Introduction

Infectious disease caused by spirochetes of the genus *Leptospira* is a veterinary and public health problem of global proportions [1], [2]. Humans and other mammals are exposed to the organism when they contact groundwater contaminated with urine from carrier animals. The disease in humans varies from a mild flu-like form to a more severe syndrome involving multiorgan failure and death [3]. Uveitis is a common complication of systemic infection in humans affecting one or both eyes [4]. In equines, infection is mainly associated with spontaneous abortion in mares and recurrent uveitis [3]. After an initial infection, some horses develop a recurrent inflammation of the uveal tract of eye (iris, ciliary body and choroid), known as equine recurrent uveitis (ERU) or ‘moon blindness’. First described in 1819 by James Wardrop as a “specific inflammation” of uveal origin, it is the most common cause of blindness in horses worldwide [5], [6] with a prevalence of approximately 8–10% in the United States [7]. Onset of the disease is usually acute with variable degrees of severity and duration. The acute phase is followed by a quiescent phase of no or low inflammation [8]. Subsequent recurrence of inflammation results in pronounced lesions with guarded prognosis for preservation of visual acuity [8], [9], [10], [11]. The Appaloosa breed and horses with MHC class I haplotype ELA-*A9* have been observed to be at increased risk of developing uveitis [12], [13].

*Leptospira interrogans* serovar Pomona is the most common and well-documented infectious cause of ERU in the United States [14]. Its association with pathogenic leptospires has been well established by presence of high titers of leptospiral agglutinins in the blood and aqueous humor [15], [16], by isolation of *Leptospira* from ocular fluids [17], [18] and the detection of leptospiral DNA by polymerase chain reaction in vitreous humor of uveitic horses [17]. Initial evidence of the association was provided by Morter et al. [19] when they induced uveitis in ponies by subcutaneous injection of guinea pig blood containing live *L. interrogans* serovar Pomona. The resulting ocular pathology in experimental ponies was found to be similar to that of spontaneous cases of *Leptospira*-associated ERU.

By using ERU uveitic fluids to screen a lambda phage library of *L. interrogans*, we identified leptospiral lipoproteins, LruA and LruB, associated with recurrent uveitis in horses [20]. Uveitic equine eye fluids contained significantly higher levels of immunoglobulin A (IgA) and IgG specific for LruA and LruB than did companion sera, indicating strong local antibody responses. Moreover, monospecific antiserum to LruA and LruB reacted with extracts of equine ocular tissue. In the present study we have examined the reactivity of LruA- and LruB-antiserum with sections of lens and retinal tissue and identified the ocular proteins involved in the interaction. In addition, the significance of the identified autoantigens was assessed by measuring their immuno-reactivities in eye fluids of uveitic and healthy animals.

## Materials and Methods

### Ethics statement

All animals were handled in strict accordance with relevant national and international guidelines, and all animal work was approved by the University of Kentucky Institutional Animal Care and Use Committee (IACUC#2009-0477).

### Eye fluids and eye tissue extracts

Eye fluids and companion sera from horses of varied age, breed, and origin were obtained from a commercial horse slaughter plant in North America. Eyes with gross evidence of uveitis were enucleated after slaughter, and aqueous humor was removed with a 10-ml syringe and stored at −20°C. The eyes were placed in 10% formaldehyde for subsequent embedding, sectioning, and staining with hematoxylin and eosin for histological examination. Eye fluids and sera were assayed for antibodies to serovars Pomona, Canicola, Icterohemorrhagiae, Hardjo, Bratislava, and Grippotyphosa in the microscopic agglutination test (MAT) [20]. Eye fluids and sera from each horse were also tested by ELISA using recombinant antigens LigA, Lk73.5 and Qlp42). Extracts were prepared from the ciliary body, cornea, lens, and retina of a normal eye from a young horse serologically negative for *Leptospira*, as described by Parma et al., 1985 [21].

### Recombinant protein and antiserum

Identification, cloning, and expression of recombinant LruA and LruB has been described previously [20]. Briefly, following PCR amplification of chromosomal DNA of *L. interrogans* serovar Pomona type kennewicki (JEN4) with gene-specific primers, amplicons were inserted into pET-15b (Novagen, Madison, WI). Recombinant plasmids were transformed into *Escherichia coli* BL21 (DE3) (Novagen, Madison, WI), and recombinant His-tagged proteins were isolated and their purity tested as previously described [20]. Three New Zealand white rabbits were immunized to obtain polyclonal antiserum directed against recombinant LruA.

### Immuno-fluorescence assay (IFA)

Lenses were dissected from the eyes of three healthy horses immediately after euthanasia, frozen in liquid nitrogen, stored at −70°C, and later embedded in tissue freezing medium for mounting in a Tissue-Tek (Miles, Elkhart, IN) cryostat. Sections (8–10 µ) were placed on glass slides treated with 2% solution of 3-aminopropyltriethoxysilane in acetone. The sections were fixed in acetone at 20°C for 20 min followed by two washes with phosphate buffer saline (PBS, pH 7.4) for 5 min each. Blocking was performed using 2% bovine serum albumin (BSA; Sigma, St. Louis, MO) in PBS for 30 min. Sections were again washed thrice with PBS and incubated with 1∶100 polyclonal rabbit antiserum or pre-immune serum overnight at 4°C in a humidifying chamber. Sections were washed three times for 5 min each and subsequently incubated with 1∶250 dilution of FITC conjugated goat anti-rabbit IgG (Invitrogen, Carlsbad, CA) for 1 h at room temperature in a humidifying chamber. Slides were mounted in a mounting medium containing anti-fading reagent Mowiol (EMD Chemicals, Gibbstown, NJ) and screened by epifluorescence microscopy (Axioscope-20; Zeiss, Thornwood, NY, USA) and image analysis was carried out using the QUIPS-XL and QUIPS-AKS system (Vysis, Downer's Grove, IL, USA). The same IFA protocol as above was used for testing lens tissues obtained from two healthy sheep.

### Two dimensional polyacrylamide gel electrophoresis

Lenticular and retinal aqueous extracts were separated by two-dimensional polyacrylamide gel electrophoresis (2-D PAGE) using MultiPhor-II system (GE Healthcare, Piscataway, NJ). Briefly, the aqueous extracts were subjected to isoelectric focusing using precast IPG strips (Bio-Rad, Hercules, CA) for 3000 V· h (500 V, 6 h, 10°C). Strips were then equilibrated and subjected to conventional sodium dodecylsulfate-12.5% polyacrylamide gel electrophoresis (SDS-PAGE). Gels were either stained with SYPRO Ruby (Invitrogen) or transferred to nitrocellulose membranes for immunoblot analysis with LruA- or LruB-directed antiserum. Immunoblot positive protein spots were extracted from gels and analyzed by matrix-assisted laser desorption ionization-time-of-flight (MALDI-TOF) mass spectrometry (University of Louisville Mass Spectrometry Core Laboratory, Louisville, KY). Spectrometry outputs were compared with known sequences using Mascot (Matrix Science, Boston, MA).

### Eye protein antibody assays

Recombinant human α-crystallin B (Abcam, Cambridge, MA), purified vimentin from bovine lens (Sigma) or human recombinant β-crystallin B2 (Abnova) were separated by SDS-PAGE, transferred to a nitrocellulose membrane and blocked with 5% nonfat dry milk in Tris-buffered saline (20 mM Tris, 150 mM NaCl, 0.05% Tween 20, pH 7.5). Membranes were incubated with LruA or LruB-antiserum (1∶400) followed by incubation with protein G conjugated to horseradish peroxidase (Zymed, San Francisco, CA). Membranes were developed with the SuperSignal West Pico enhanced chemiluminescence substrate (Pierce), and bands were visualized with BioMax Light film (Kodak).

ELISA measured alpha-crystallin B, vimentin and β-crystallin B2 antibody levels in leptospiral uveitic and normal eye fluids as described previously [20], [22]. Briefly, ELISAs were performed in Maxisorp 96-well plate wells (Nalge-Nunc, Rochester, NY) coated with 200 ng human recombinant alpha-crystallin B (Abcam), purified vimentin from bovine lens (Sigma) or human recombinant β-crystallin B2 (Abnova) followed by blocking with 5% nonfat dry milk. Uveitic and normal eye fluids (1∶100) were added and incubated for 1 h at 37°C. Bound antibodies were detected using HRP conjugated Protein G (1∶4000; Zymed, San Francisco, CA). Plates were developed using ready-to-use 3,3′,5,5′-tetramethyl benzidine substrate solution (1-Step Turbo TMB-ELISA, Thermo Scientific, Rockford, IL). Reactions were stopped by addition of 2N H_2_SO_4_, 50 µl/well. Absorbance was read at 450 nm in a Spectramax plate reader using SoftMax Pro (Molecular Devices, Sunnyvale, CA). Statistical analyses were performed using Student's t-test assuming unequal variances.

## Results

### Cross-reactivity of LruA and LruB with equine lens and retina, respectively

**Figure 1:**
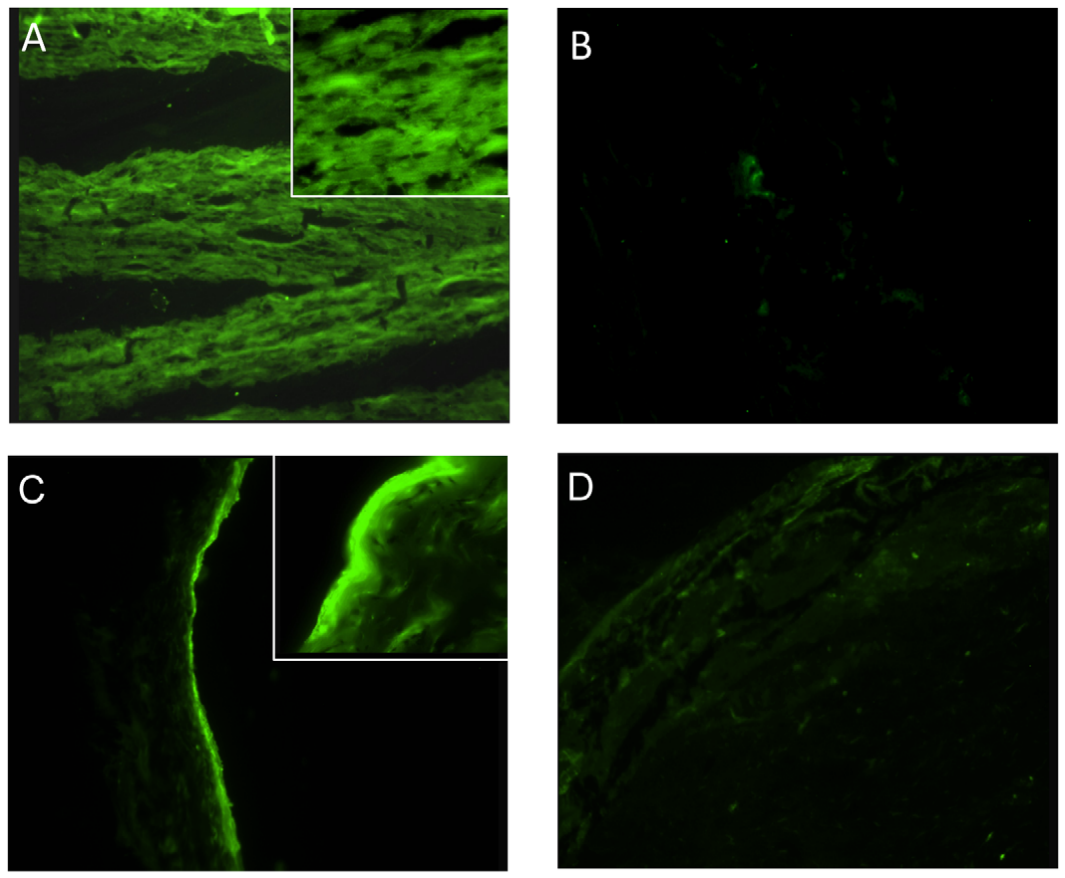
LruA and LruB-antiserum reacts with lenticular and retinal tissues. (**A**) Photomicrographs showing uniform homogeneous fluorescence in a section of equine lens incubated with LruA-antiserum (1∶100) but not with preserum (**B**) (×100). (**C**) Photomicrographs showing a positive fluorescence in a frozen section of equine retina incubated with LruB-antiserum (1∶100) but not with pre-immunization serum (**D**) (×100). Inset (×400).

In a previous study [20] LruA-antiserum was shown to recognize a ∼22 kDa protein in lens extract and a ∼65 kDa protein in ciliary body extract. In the same work, LruB-antiserum reacted with a ∼30 kDa band in retinal extract. To further examine the observed cross-reactivity between equine ocular tissue and LruA and LruB specific antisera, immunofluorescent assays were performed. Frozen lenticular and retinal tissue sections (8–10 µ) were fixed, blocked and incubated with antiserum or preserum to LruA and LruB (diluted 1∶100). The lens fibers showed uniform homogenous pattern of fluorescence when incubated with LruA-specific antiserum but not with normal rabbit serum (**Figure 1A and B**).

Similarly, fluorescence was observed when equine retinal tissue sections were incubated with LruB-specific antiserum but not with normal rabbit serum (**Figure 1C and D**). The positive fluorescence seen in retinal tissue incubated with LruB-antiserum was restricted to one or more deeper retinal layers, which include the inner limiting membrane, layer of nerve fiber and may be the ganglion cell layer, in contrast to a diffused positive fluorescence seen in lens tissue sections incubated with LruA-antiserum. In addition, sclera and choroid were devoid of any fluorescence in the same tissue section (**Figure 1** **)**.

Similar results were obtained with lenticular tissues from a healthy sheep. Sections of sheep lens incubated with LruA-antiserum showed positive fluorescence but not when these sections were incubated with pre-immune serum (not shown). The reactivities of equine and sheep lenticular tissue sections with LruA-antiserum indicated that the observed interaction is not unique to equines lens.

### Identification of lenticular and retinal proteins cross-reactive with LruA and LruB

**Figure 2:**
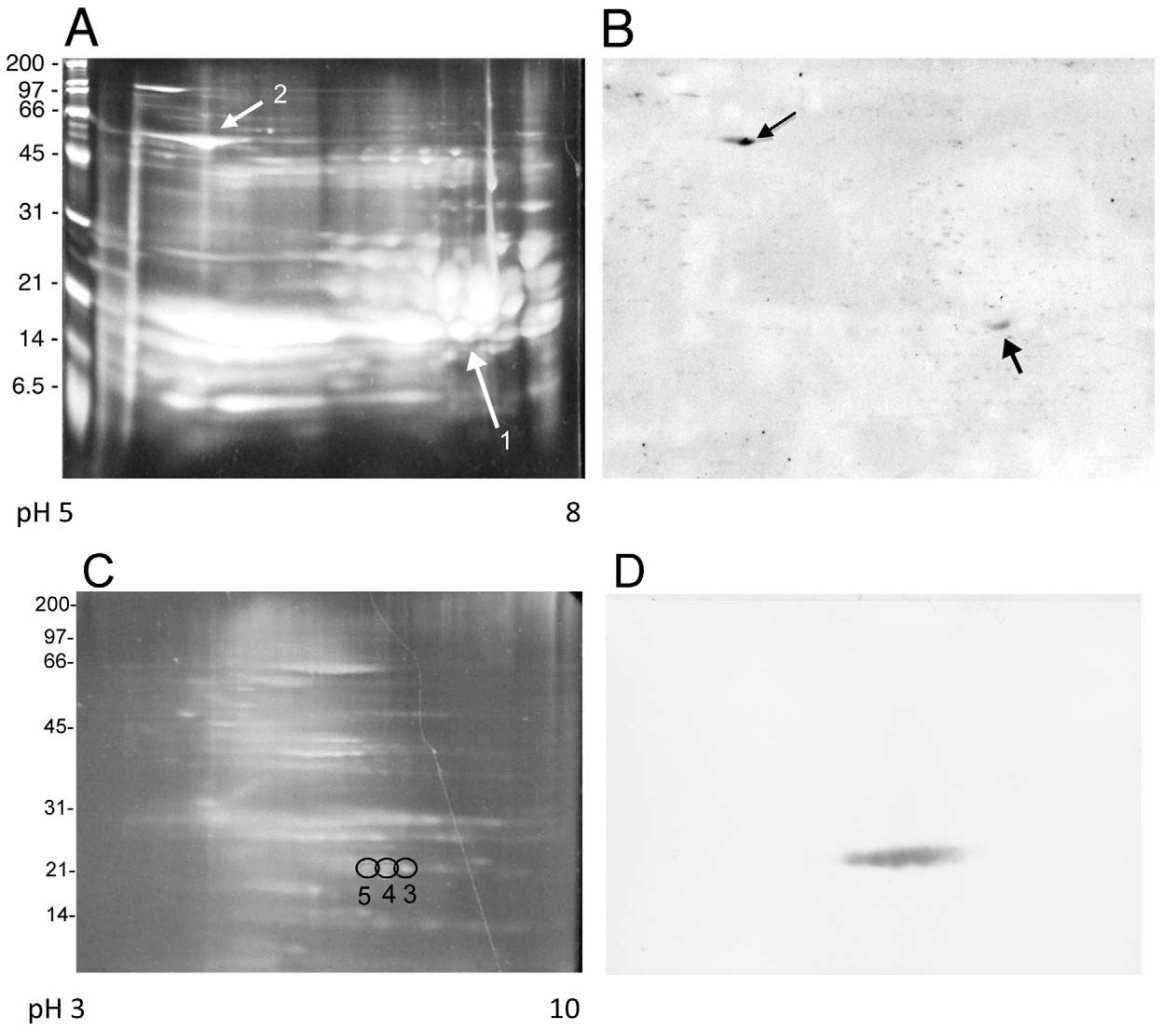
Two-dimensional electrophoretic analysis of proteins in equine lenticular and retinal tissue extracts. (**A**) Lens extract separated on a polyacrylamide gel stained with the fluorescent dye SYPRO-Ruby. (**B**) Lens proteins transferred from a second gel to nitrocellulose membrane and blotted with LruA-antiserum. The arrowheads indicate the protein spots excised from the stained gel for analysis by mass spectrometry. (**C**) Retinal extract separated on a polyacrylamide gel stained with SYPRO-Ruby. (**D**) Retinal proteins transferred from a second gel to nitrocellulose membrane and blotted with LruB-antiserum. Three protein spots (numbered 3, 4 and 5) were excised from the stained gel for analysis by mass spectrometry. Results of mass spectrometric analyses are tabulated in Table 1.

To identify the eye protein(s) recognized by LruA-directed antibodies, proteins in equine lenticular extract were separated on two-dimensional polyacrylamide gels, transferred to nitrocellulose membranes and probed with LruA-specific antiserum (**Figure 2 A and B**). LruA-antiserum recognized protein spots with apparent molecular masses of approximately 20 and 60 kDa. The immunoblot was aligned with the stained gel, to locate the corresponding protein spots which were then subjected to mass spectrometric analysis. The 20 and 60 kDa protein spots were identified as α-crystallin B and vimentin, respectively (**Table 1**) with 66% and 44% coverage (not shown). Alpha-crystallin B and vimentin have molecular masses of 20188 Daltons and 53727 Daltons, respectively. Attempts to identify ciliary body protein(s) reactive to LruA-specific antiserum by this method have not yet been successful.

**Table 1:** Identification of ocular proteins cross-reactive with LruA and LruB antiserum (Figure 2) by mass spectrometry.

Protein Spot	Identified Protein	Accession Number	Predicted Mass*
Spot 1	Alpha-crystallin chain B	CYBOAB	20024
Spot 2	Vimentin	VIM_BOVINE	53752
Spot 3	Beta-crystallin chain B2	Q2 LEC2_CANFA	23318
Spot 4	Beta-crystallin chain B2	Q2 LEC2_CANFA	23318
Spot 5	Beta-crystallin chain B2	Q2 LEC2_CANFA	23318

*Peptide masses were analyzed using the MASCOT Database search engine v1.9 (www.matrixscience.com) (Matrix Science Ltd.).

Similarly, LruB-antiserum recognized three spots of retinal proteins (**Figure 2C and D**), which were identified by mass spectrometry to be β-crystallin B2 (**Table 1**).

### LruA-directed antiserum recognizes human eye lens α-crystallin B and vimentin

**Figure 3:**
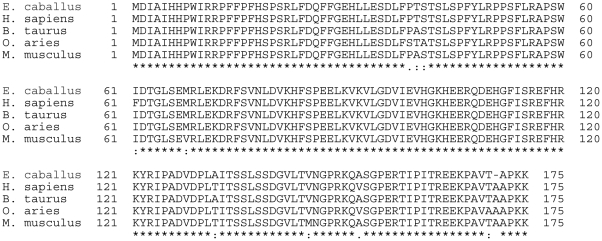
Multisequence alignment of alpha-crystallin B of horse (*Equus caballus*), man (*Homo sapiens*), cow (*Bos taurus*), sheep (*Ovis aries*) and mouse (*Mus musculus*) using T-COFFEE Version 5.05 [http://www.tcoffee.org].

**Figure 4:**
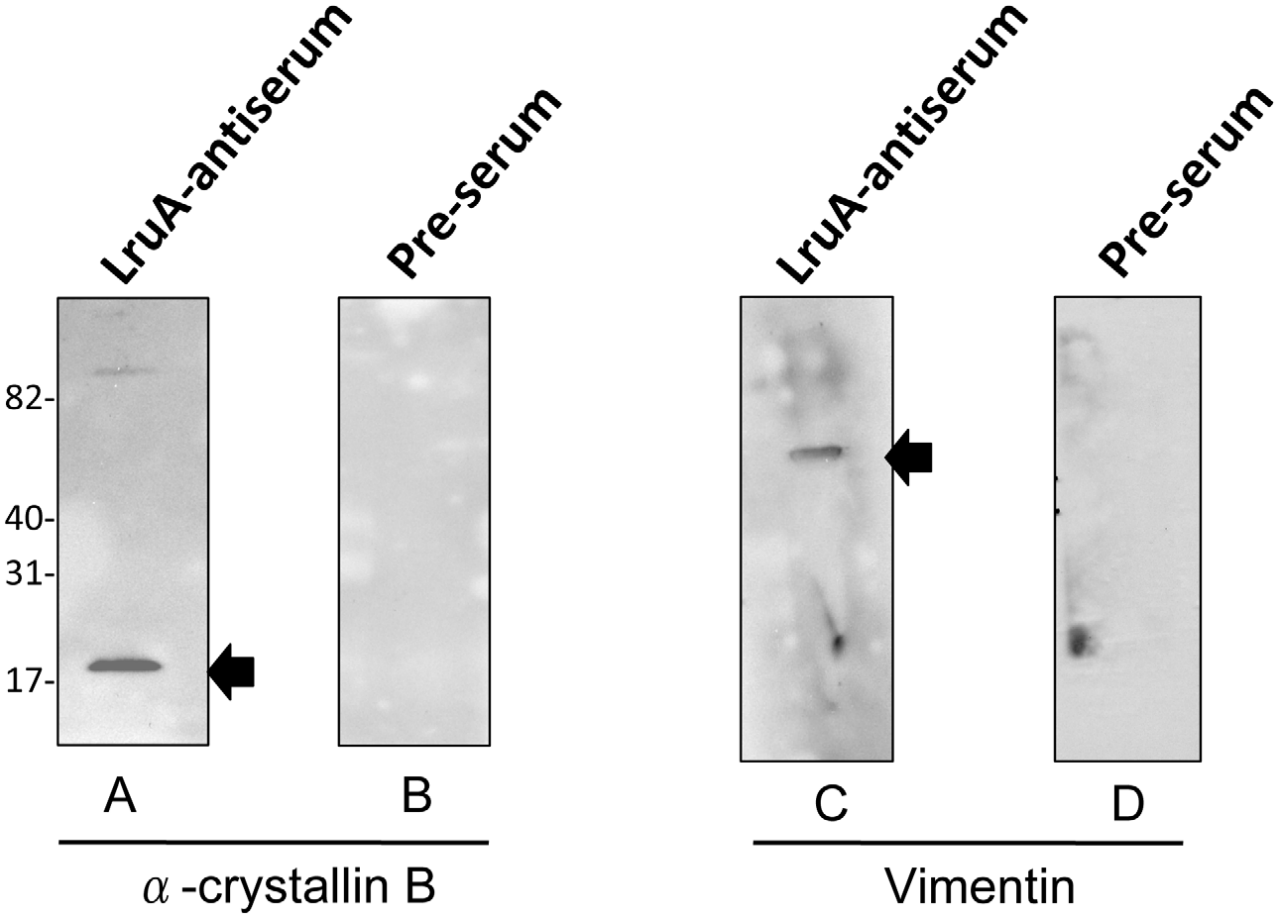
LruA antiserum reacts with recombinant human alpha-crystallin B and purified vimentin. (**A**) Immunoblot showing reactivity of LruA-specific antiserum (1∶400) with recombinant human alpha-crystallin B (1µg). (**B**) Pre-immune serum (1∶400) did not react with this protein. (**C**) Immunoblots showing reactivity of LruA-antiserum (1∶400), but not the pre-immunization serum (**D**), with purified vimentin (1µg). Molecular mass markers are indicated in kilodaltons.

Mammalian α-crystallin B protein sequences are highly conserved across species (**Figure 3**). Therefore, purified recombinant human α-crystallin B was used in immunoblot analyses to confirm α-crystallin B as a cross-reacting antigen. LruA-directed antiserum, but not the pre-immune serum, reacted with recombinant α-crystallin B, indicating that this lenticular protein is indeed the cross-reacting antigen (**Figure 4A and B**).

The amino acid sequence identity between equine and bovine vimentin is 91% (not shown). So, purified vimentin from bovine lens was used in immunoblot to examine its reactivity to LruA-directed antiserum. LruA-directed antiserum but not the pre-immune serum, reacted with purified vimentin in an immunoblot (**Figure 4C and D**).

### LruB-directed antiserum recognizes human β-crystallin B2

**Figure 5:**
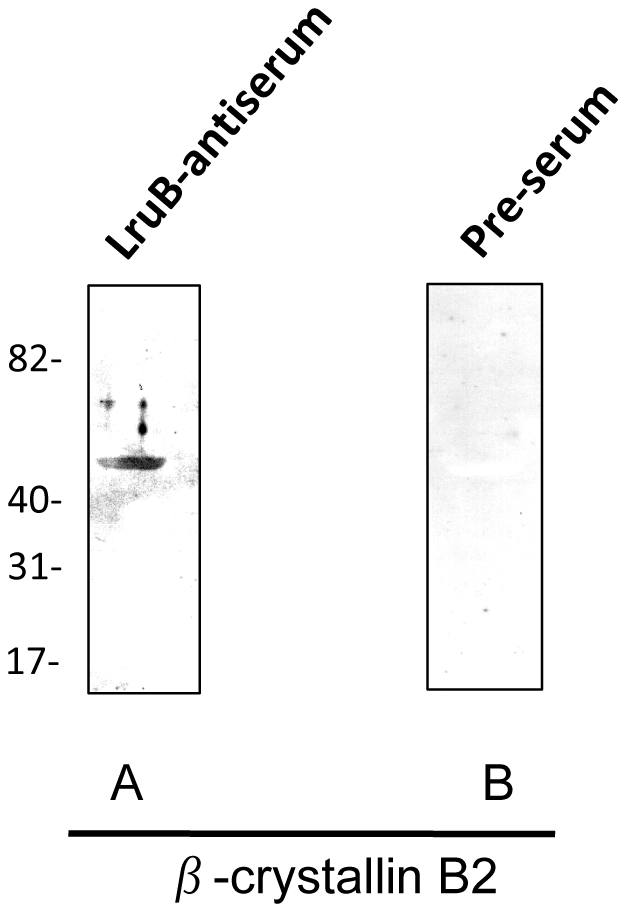
LruB antiserum reacts with human β-crystallin B2. (**A**) Immunoblots showing reactivity of LruB-antiserum (1∶400) with recombinant human β-crystallin B2(1µg). (**B**) Pre-immune serum (1∶400) did not react with β-crystallin B2. Molecular mass of recombinant β-crystallin B2 is 50.4 kDa. Molecular mass markers are indicated in kilodaltons.

Recombinant human β-crystallin B2 was used in immunoblot analyses to confirm β-crystallin B2 as a cross-reacting antigen. LruB-directed antiserum, reacted with recombinant β-crystallin B2, indicating that this lenticular protein is indeed the cross-reacting antigen (**Figure 5A**). Pre-immunization serum did not react with β-crystallin B2 (**Figure 5B**).

### Antibodies directed to alpha-crystallin B, vimentin and β-crystallin B2 in uveitic fluids

**Figure 6:**
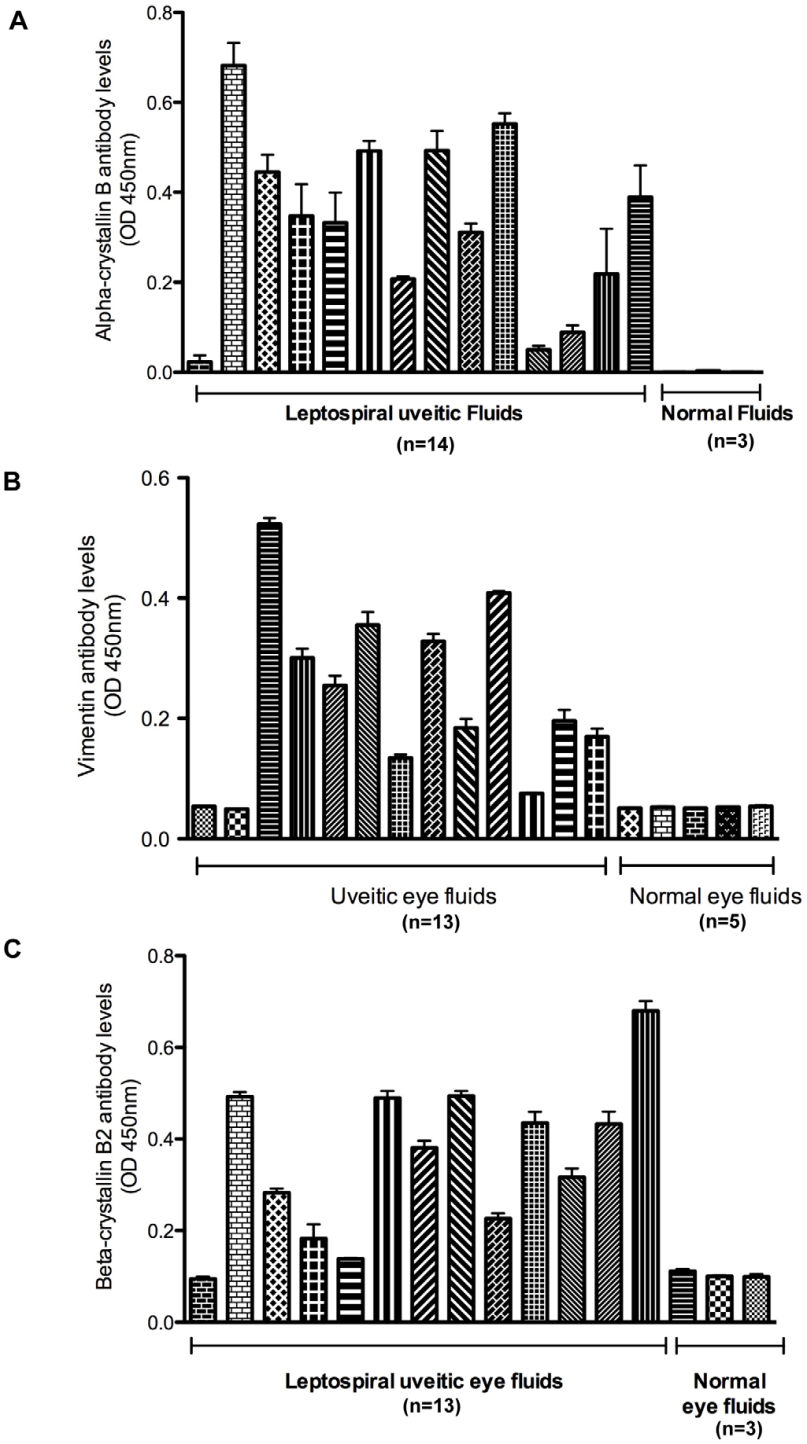
Uveitic eye fluids contain antibody to alpha-crystallin B, vimentin and β-crystallin B2. ELISA results showing significantly higher antibody levels of alpha-crystallin B (**A**), vimentin (**B**) and β-crystallin B2-antibodies (**C**) in uveitic eye fluids. ELISA plate wells were coated with 200 ng of each eye protein. After blocking, wells were sequentially incubated with uveitic or normal eye fluids diluted 1∶100 and HRP-conjugated protein G (1∶5000). Plates were developed using 3,3′,5,5′-tetramethyl benzidine substrate solution and absorbance measured at 450 nm. Presented data is a representative of at least two independent experiments with 3 or more repeats. Error bars indicate standard deviation.

The biological significance of α-crystallin B, vimentin and β-crystallin B2 as a cross-reacting antigen was investigated by examining antibody levels against these lenticular or retinal proteins in eye fluids obtained from clinical cases of leptospiral uveitis and healthy controls. ELISA was performed using recombinant α-crystallin B, purified vimentin or recombinant β-crystallin B2 as coating antigens. Antibody levels to α-crystallin B, vimentin and β-crystallin B2 (**Figure 6**) were found to be significantly elevated in uveitic compared to normal eye fluids (p<0.001).

## Discussion

The pathogenesis of leptospiral uveitis is currently under investigation and several possible mechanisms have been proposed [6], [9], [12], [17], [20], [21], [23], [24], [25], [26], [27], [28]. How leptospires survive in the eye, causing breach of the ocular immune privilege and initiation of pro-inflammatory changes, is not understood. Although direct *Leptospira*-mediated injury to eye structures is possible, a growing body of evidence suggests that autoimmune responses to ocular tissue components play a significant role in pathogenesis [6], [9], [12], [17], [20], [21], [22], [23], [24], [25], [26], [27], [28]. Parma et al. [21] demonstrated reactivity of anti-equine cornea antibodies with *Leptospira* and binding of *Leptospira* and cornea specific antibodies to equine cornea [21]. Subsequently, an antigenic relationship between equine lens and leptospires was proposed by the same group [27]. Electron microscopic studies revealed that the antigenic protein of *L. interrogans* that shares epitopes with equine cornea and lens is not exposed on the outer surface of leptospires [28]. However, in those studies, specific leptospiral and/or ocular proteins involved in the antigenic relationship were not identified. In this study, we have shown that the lenticular proteins, α-crystallin B and vimentin, cross-react with LruA and retinal protein, β-crystallin B2, cross-reacts with LruB confirming our previous observations of reactivity of LruA and LruB antibodies with equine lens and retina, respectively.

Alpha-crystallin B and vimentin are critical for maintaining lens clarity and thus visual acuity [29]. Alpha-crystallin is the principal constituent of the lens and acts as a molecular chaperone that keeps other lens proteins from precipitating [30]. Disruption of this function may lead to impairment of light refraction and potentially vision. Alpha-crystallin B is a 175-amino acid small heat shock protein and shares high interspecies sequence homology (**Figure 3**). Its involvement in several disease states including uveitis, Alexander disease, Alzheimer's, Creutzfeldt-Jacob disease and multiple sclerosis are under investigation [31], [32], [33]. In addition to lens and central nervous system (CNS), it is also present in many other tissues including skeletal muscles and kidney epithelial cells.

Vimentin is an important structural determinant in the human lens cell and is mainly expressed in the epithelium of the lens. In a previous study, high expression of vimentin was negatively correlated with the normal differentiation of the lens fibers. In that study, animals developed pronounced cataract and extensive lens degeneration as a result of impairment of lens fiber cell differentiation [34]. A study on expression of vimentin in lens epithelium of age-related cataract suggested that damage to the lens epithelial cells might initiate a decrease in vimentin expression leading to degradation of the lens cytoskeleton [35]. Recently, small interfering RNA (siRNA) mediated downregulation of human pigment epithelium-derived factor (PEDF) expression in primary human lens epithelial cells was shown to result in a decrease in the expression of vimentin and increase of α-crystallin B expression [29]. Interestingly, serum and ocular levels of PEDF have been shown to decrease in uveitic horses, but not the normal horses [36], [37].

Beta-crystallin B2 is present in lens and non-lenticular tissues, including the retina. The appearance and accumulation of beta-crystallin B2 in neural retina coincides with its functional maturation [38]. Recently, antibodies against α-crystallin A, α-crystallin B and β crystallin B1 were found to be significantly elevated in uveitis patients and seroreactivity was found to be significantly associated with cortical cataract [39]. In another study, Çelet and colleagues [31] reported an elevated humoral response to α-crystallin B in neuro-Behçet's disease and Guillain-Barré syndrome. We recently demonstrated that LruA and LruB were recognized by antibodies from Behçet's and Fuchs uveitis patients, without any evidence of those patients having been exposed to *Leptospira* [22]. Both of these diseases are believed to be autoimmune diseases [22], [40], [41], [42], [43], [44], [45]. In the same study, we also observed an association in humans between high levels of antibodies recognizing LruA and LruB and the presence of cataract [22]. The high levels of antibodies cross-reactive with LruA and LruB in patients with Fuchs or Behçet's uveitis, and the strong association of LruA and LruB antibodies with cataract could be due to increased levels of antibodies to the common autoantigens, α-crystallin B, vimentin and β-crystallin B2, in those diseases. Also, elevated levels of LruA- and LruB-antibodies in sera of human patients with leptospiral uveitis [22] and reactivity of LruA- and LruB-antiserum with human alpha-crystallin B and β-crystallin B2 suggest a similar phenomenon in human leptospiral uveitis. We are presently pursuing those hypotheses to determine the causes of leptospiral and non-leptospiral uveitis.

A linear amino acid similarity or a conformational homology between microbial and host proteins is a potential basis for molecular mimicry. The limited linear amino acid similarity between these leptospiral proteins and their respective cross-reacting ocular proteins (not shown) suggests similarities at the conformational level. Studies to identify the cross-reactive epitopes are underway.

In conclusion, we have identified two lens proteins and a retinal protein that react with antiserum directed against LruA and LruB, leptospiral proteins expressed in uveitic eyes. The presence of antibodies recognizing α-crystallin B, vimentin and β-crystallin B2 in uveitic, but not normal eye fluids, strongly suggests a role for these antibodies in *Leptospira*-associated recurrent uveitis. In the immune privileged ocular environment, it is likely that the early phase of leptospiral infection involves a non-inflammatory immune responses specific for LruA and LruB. Resulting antibodies may interact with cross-reacting proteins in lens and retinal tissues and may therefore initiate a process of desequestration of these ocular antigens, and possibly other components. How early after an initial infection this interaction results in development of the changes in eye, and what other pro-inflammatory changes, if any, are required remains to be determined.
